# Superconducting flux concentrator coils for levitation of particles in the Meissner state

**DOI:** 10.1093/pnasnexus/pgag072

**Published:** 2026-03-16

**Authors:** Robert Smit, Martijn Janse, Eli van der Bent, Thijmen de Jong, Kier Heeck, Jaimy Plugge, Tjerk Oosterkamp, Bas Hensen

**Affiliations:** Leiden Institute of Physics, Leiden University, P.O. Box 9504, Leiden 2300 RA, The Netherlands; Leiden Institute of Physics, Leiden University, P.O. Box 9504, Leiden 2300 RA, The Netherlands; Leiden Institute of Physics, Leiden University, P.O. Box 9504, Leiden 2300 RA, The Netherlands; Leiden Institute of Physics, Leiden University, P.O. Box 9504, Leiden 2300 RA, The Netherlands; Leiden Institute of Physics, Leiden University, P.O. Box 9504, Leiden 2300 RA, The Netherlands; Leiden Institute of Physics, Leiden University, P.O. Box 9504, Leiden 2300 RA, The Netherlands; Leiden Institute of Physics, Leiden University, P.O. Box 9504, Leiden 2300 RA, The Netherlands; Leiden Institute of Physics, Leiden University, P.O. Box 9504, Leiden 2300 RA, The Netherlands

**Keywords:** magnetic levitation, superconductors, NV magnetometry, flux trapping

## Abstract

Magnetic levitation of superconductors is a promising platform to study quantum mechanics in the large-mass limit. One major limitation is the weak trapping potential, which results in low vibrational eigenfrequencies and increased susceptibility to low-frequency noise. While generating strong magnetic fields is relatively straightforward, creating a tightly confined harmonic potential—essentially achieving a large magnetic field gradient—remains a significant challenge. In this work, we demonstrate a potential solution using superconducting cores that concentrate magnetic flux into arbitrarily small volumes. We show the ability to trap superconducting particles using an anti-Helmholtz coil configuration incorporating these cores. However, we observe rapid damping of the levitated particle motion due to flux trapping within the cores, occurring once the lower critical field is exceeded locally. To investigate this mechanism, we employ diamond nitrogen-vacancy center magnetometry and detect substantial remanent fields persisting after high-current operation of the coils. Finally, we discuss possible strategies to mitigate this effect and improve the levitation properties.

Significance Statement Superconducting particles levitated in magnetic fields offer a unique route to isolating massive objects from their environment, enabling exploration of quantum behavior at macroscopic scales. A major challenge has been achieving stable, strong confinement in millikelvin environments, while minimizing noise. We demonstrate a superconducting flux concentrator trap that meets this need by amplifying the magnetic field within the trap through flux concentration. Subsequently, we show how flux trapping influences particle dynamics using diamond-based magnetometry to quantify local fields. This platform paves the way for precision sensing, fundamental quantum experiments, and technologies that exploit levitated superconductors.

## Introduction

The levitation of particles is a powerful and widely employed technique for isolating objects from their environment, thereby minimizing dissipative interactions (1). The achievement of a long-lived and large-mass motional quantum state would be a milestone towards addressing fundamental questions in physics, including those concerning wave function collapse models (2) and the interplay between quantum mechanics with gravity (3, 4). Beyond theoretical implications, levitated particles also serve as highly sensitive platforms for detecting gravitational forces (5), torques (6), and even individual nuclear decay events (7).

The current state-of-the-art in particle levitation is the optical tweezer, which has realized significant milestones over the past decade (8, 9). A particular achievement is the cooling of the motion of a levitating silica nanoparticle to the quantum ground state, first along a single vibrational mode (10, 11) and later in two modes simultaneously (12). Despite these achievements, optical traps inherently suffer from heating via photon absorption (13) and scattering (14), a limitation that becomes increasingly severe for larger, more massive particles, particularly for those approaching the 1 μm scale, where gravitational effects are expected to become relevant (3).

Alternative levitation techniques, based on electric and magnetic fields, offer potential pathways to circumvent these limitations. According to Earnshaw’s theorem, stable levitation of charged or magnetic objects using static fields alone is impossible, necessitating dynamic potentials such as the rotating saddle potential in electric (15) and magnetic Paul traps (16, 17). However, static magnetic levitation is achievable for diamagnetic particles that acquire an induced dipole in an applied magnetic field (18, 19).

Superconducting particles represent an ideal diamagnetic system, perfectly repelling external magnetic fields by the Meissner effect (20). Magnetic confinement of such particles has been demonstrated using anti-Helmholtz coil configurations with both superconducting wire loops (21) and planar on-chip coils (22). However, the achievable eigenfrequencies of motion within a magnetic trap remain significantly lower than those possible in optical traps. A clear route to increasing them involves higher coil currents, but this approach introduces substantial technical difficulties when operating in a millikelvin environment, more so when including vibration isolation systems (21). In this work, we explore an alternative strategy: we employ superconducting flux concentrators to locally enhance the magnetic field gradient, thereby enabling stable levitation of superconducting particles without resorting to impractically large currents. Furthermore, we investigate potential dissipation mechanisms by incorporating nitrogen-vacancy (NV) center defects as nanoscale magnetic field sensors. Through this study, we provide both a promising route towards enhanced magnetic levitation systems and a detailed characterization of the associated technical challenges.

## Results

### The flux concentrator trap

**Fig. 1.**
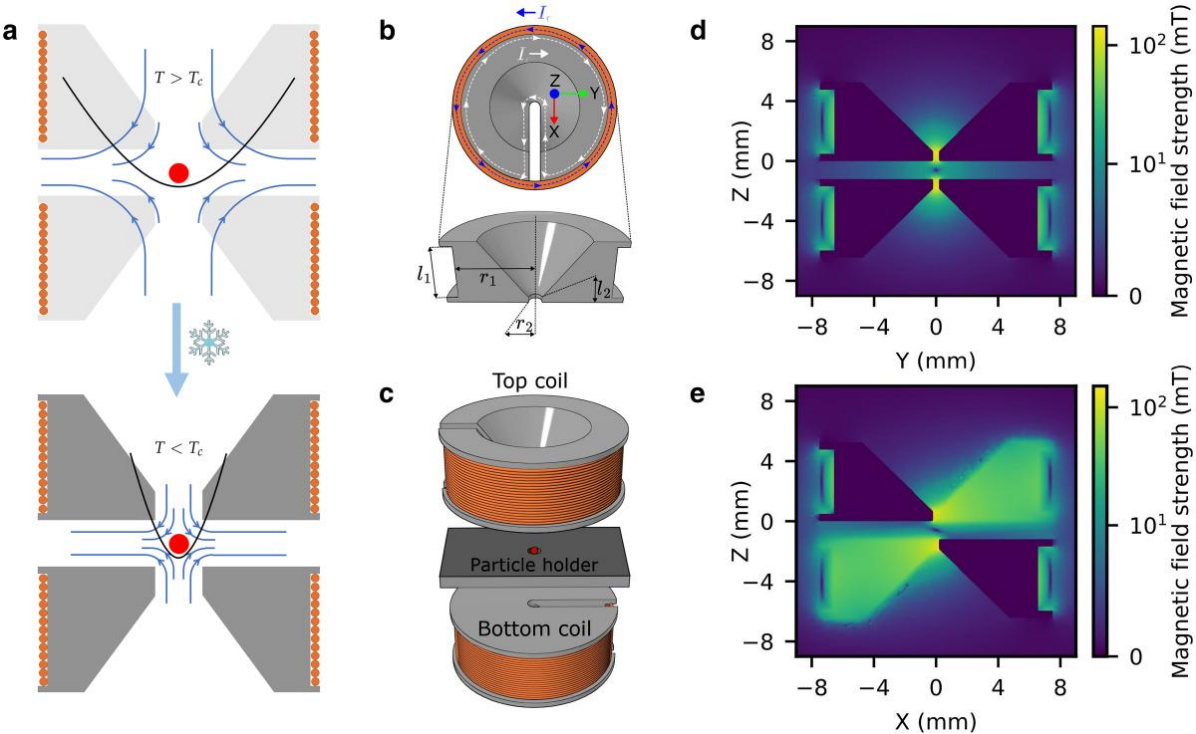
Flux concentration to enhance trap stiffness for Meissner levitation in anti-Helmholtz configuration. a) Two coils create a quadrupole magnetic field (field lines) resulting in a harmonic confinement potential (parabola) for a superconducting particle (center dot). When the core enters its superconducting state from $T<T_{c}$ onward, the magnetic field flux lines of the coil are concentrated in the slit due to the Meissner effect, enhancing the magnetic field gradient and thus the stiffness of the trapping potential. b) Equivalently, a current ($I_{\mathrm{coil}}$) through the outer wound coil induces an equal shielding current that revolves around an inner loop, leading to local concentration of the magnetic field. The dimensions of the cores in the experiments are $l_{1}=4.5\;\mathrm{mm}$, $l_{2}=0.45\;\mathrm{mm},r_{1}=6.57\;\mathrm{mm}$, and $r_{2}=0.2\;\mathrm{mm}$. c) A set of these identical coils, placed in anti-Helmholtz configuration. The spacer is a PEEK plate with the superconducting particle inside. d, e) The simulated potential minimum of the magnetic field for projections on the YZ and XZ axis, according to the axes system shown in (b). The simulations correspond to an applied current of 1 A through both coils.

The earliest demonstrations of magnetic flux focusing or concentration involved coils wrapped around electrically conductive materials. These flux concentrators were originally developed to produce high transient magnetic fields (23) or to generate strong localized fields in a confined region (24). In our flux concentrator configuration, illustrated in Fig. 1a, the magnetic field produced by an outer coil induces circulating eddy currents within the core. By introducing a slit into the core, extending to its center, the induced current is forced to navigate around a narrow central path, resulting in a locally enhanced magnetic field. The absence of resistive losses and the perfect diamagnetic shielding inherent to superconductors make them highly efficient flux concentrators, a capability demonstrated using a range of superconducting materials (25, 26). While flux concentrators can dramatically increase local field strengths, the enhancement persists only until the applied field approaches the critical current of the superconducting core. For levitation applications, however, the field strength itself is less critical than the achievable magnetic field gradient. Notably, superconducting flux concentrators have recently been proposed as a means of generating large field gradients suitable for diamagnetic levitation (27). The use of superconductors to concentrate magnetic fields would, moreover, eliminate eddy current damping of a levitating particle’s motion. Beyond generating concentrated fields, similar designs have been applied to efficiently collect magnetic flux from localized areas, particularly in scanning magnetic susceptibility measurements, where they are coupled to SQUID sensors (28).

In this work, we employ machined niobium cores with dimensions listed in the caption of Fig. 1. The system can be effectively modeled as two separate coils: the outer drive coil and the inner induced current loop within the superconducting core. Assuming that the formula for idealized long solenoids applies, the amplification factor of the magnetic field maximum at the center can be approximated as $\alpha =l_{2}/l_{1}$, where $l_{2}$ and $l_{1}$ are the characteristic dimensions of the core. Based on our core geometry, we estimate an amplification factor of ∼10, a value corroborated by finite element simulations (see Fig. S8a).

To create a trapping potential suitable for levitating a superconducting particle, a pair of these flux concentrator coils is assembled in an anti-Helmholtz configuration, with their slits antialigned and stacked (Fig. 1b). A 1 mm thick PEEK spacer, sandwiched between two 100 μm cover glasses, separates the coils, leaving a cavity to hold a 50 μm diameter tin-lead (63:37) alloy particle (EasySpheres), which is expected to transition to a superconducting state below 7 K (29). The coils around the niobium cores comprise 180 turns of 100 μm niobium–titanium wire with copper cladding (SuperCon).

Finite element simulations of this double-concentrator anti-Helmholtz configuration confirm the presence of a magnetic field minimum between the two antialigned slits (Fig. 1c and d). The modeled intercoil spacing of 1.2 mm corresponds to the physical thickness of the PEEK spacer and cover glasses. Under these conditions, the calculated field gradients are ∼22, 34, and 56 T/m per ampere of current along the *X*, *Y*, and *Z* axes, respectively (see axes definitions in Fig. 1a), values comparable to those achieved with conventional superconducting anti-Helmholtz traps, both with wound wire coils and on-chip planar configurations (21, 22).

For the levitation experiments, the assembled flux concentrator trap was mounted in a copper holder and attached to the millikelvin stage of a dilution refrigerator (see Fig. S1). An optical access window allowed imaging of the XY plane of the trap using a camera, while illumination was provided from below via an optical fiber. This uniform illumination scheme minimized radiative heating of the levitated particle, enabling it to remain in the superconducting state for extended periods, up to tens of minutes. Superconducting wiring connected the coils to external current supplies, with thermal anchoring at intermediate refrigerator stages. Continuous currents up to 1.7 A could be applied, with levitation typically achieved at currents between 600 and 800 mA. Initial ring-down measurements following levitation exhibited dominant motion along the slit direction, along which the trapping potential is weakest. Subsequent adjustments to individual coil currents produced shifts in the particle’s equilibrium position along this direction (see Fig. S2), as expected due to the inclined trapping potential as observed from the simulations projected in the XZ-plane (Fig. 1c). The inclination of the potential can be avoided by matching the vertical separation of the flux concentrator coils to the radii of the coils (see Fig. S9).

The particle’s vibrational dynamics were further characterized by feeding the bottom coil of the trap with an AC current, supplied via a function generator and a 400 *Ω* current-limiting resistor, and tracking the particle motion by video analysis (see Fig. S3 for details). The primary resonance mode identified corresponded to motion along the slit direction. Other resonances were either weakly coupled, difficult to resolve optically, or resulted in the particle escaping the trap upon excitation. Consequently, our analysis focused on the x-mode (along the slit).

**Fig. 2.**
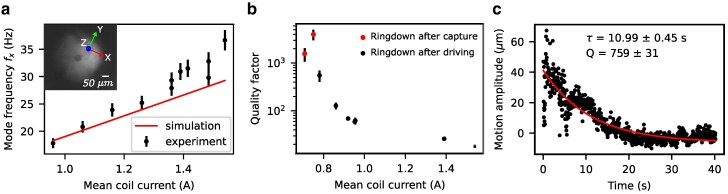
Characteristics of levitation. a) The dependence of the x-mode (in the *x*-direction, ie along the slit: see axes system in the inset picture of the levitating particle) frequency as a function of the mean current through the coils. The resonance frequency was obtained by tuning the frequency of a driving current through the bottom coil, while optically monitoring the motional amplitude with a camera. b) A set of measurements of the quality factor of the x-mode for different coil currents. The two left-most points correspond to quality factors obtained from the ringdown after trapping—thus constituting mixed modes—and the other points after driving along the slit direction. c) An example ringdown, where only the motional amplitude is included. Outlying points are explained by the difficulty of the video-tracking algorithm to track the particle’s position when the motion is relatively large. The mean coil current was ∼0.8 A.

In diamagnetic levitation systems, the vibrational mode frequencies $f_{i}$ depend on the field gradient in the direction of motion (22): $f_{i}=\nabla_{i}B\sqrt{3/8\pi \mu_{0}\rho }=\zeta_{i}\mu_{0}I/r_{2}^{2}\sqrt{3/8\pi \mu_{0}\rho }$. Here, the particle density is given by *ρ*, $r_{2}$ is the inner radius of the flux concentrator core, and $\zeta_{i}$ is a geometric factor. The equation reveals that a linear dependence with current is expected for the eigenmode frequencies. With finite element simulations we indeed find a linear relationship with current (see Fig. S10). The simulated eigenmode frequencies result in geometric factors of $\zeta_{x}=0.18,\zeta_{y}=0.36$, and $\zeta_{z}=0.53$ and reveal that the trap geometry is significantly anisotropic (20). The experimental measurements of the x-mode frequencies (shown in Fig. 2a) show an approximately linear relationship with mean coil current. However, we note that the individual measurements were not performed at the same levitation height: the ratio between the separate currents on the two coils is not constant. This may explain the offset of data points from the simulated curve at higher mean coil currents.

Interestingly, the measured resonance frequencies and damping rates were not always reproducible, varying by up to 10% depending on the preceding history of currents applied to the coils (Fig. 2b). Additionally, higher coil currents tended to degrade the system’s quality factor. The most stable and highest quality factors, reaching up to a few thousand, were observed in a freshly cooled flux concentrator that was subsequently operated at low currents only (example given in Fig. 2c). The observed quality factors compare well to those found in the work of Latorre (22). However, once higher currents were applied, the quality factors declined sharply and remained low until the entire system was thermally cycled, either by heating up the whole cryostat or by heating the flux concentrator locally with a fiber-coupled laser. These findings suggest a current-dependent operational regime for the flux concentrators, beyond which an additional or enhanced damping mechanism emerges. The likely cause involves flux penetration into the superconducting niobium cores, especially at geometric boundaries, such as the sharp edges of the slit. Simulations indicate that local magnetic fields can exceed 350 mT at the maximum applied current of 1.7 A, which is significantly higher than the lower critical field $B_{c,1}$ for high-quality niobium, reported at 173.5 mT (30). Given this, flux entry into the superconducting cores is probable at elevated currents, leading to increased dissipation: the time-varying magnetic field caused by particle motion exerts Lorentz forces on weakly pinned flux vortices, causing them to move from site to site (31, 32). This flux creep draws energy from the particle’s motion, thus reducing mechanical quality factors.

### NV center magnetometry

To investigate local magnetic field distributions and potential flux trapping within the flux concentrators, we employed NV center defects in diamond as nanoscale magnetic field probes. These sensors allow for spatially resolved, noninvasive measurements of magnetic fields within the trapping region, allowing for magnetic field mapping without having to move the magnetic field sensor in a cryogenic environment. The NV center in diamond is a point defect consisting of a nitrogen atom substituting for a carbon atom, adjacent to a vacant lattice site. In its negatively charged form, denoted NV${\;}^{-}$, the defect possesses a triplet ground electronic state with three spin projections: $m_{s}$ = |0⟩ and |±1⟩. Absorption of a photon preserves the spin state, but upon decay back to the ground state, radiative emission is more probable from the |0⟩ state than from the |±1⟩ states. This is because the |±1⟩ states preferentially decay through intersystem crossing to a metastable singlet state. The resulting imbalance in radiative pathways enables optical detection of the spin state via differences in fluorescence intensity. Transitions between spin states can be driven using resonant microwave fields, where a transition from |0⟩ to either |−1⟩ or |+1⟩ reduces the average fluorescence—a technique known as optically detected magnetic resonance (ODMR). The system’s suitability for magnetometry arises from the electron spin Hamiltonian (neglecting hyperfine interactions):


(1) $$H/\hbar =DS_{z}^{2}+\gamma S\cdot B.$$

**Fig. 3.**
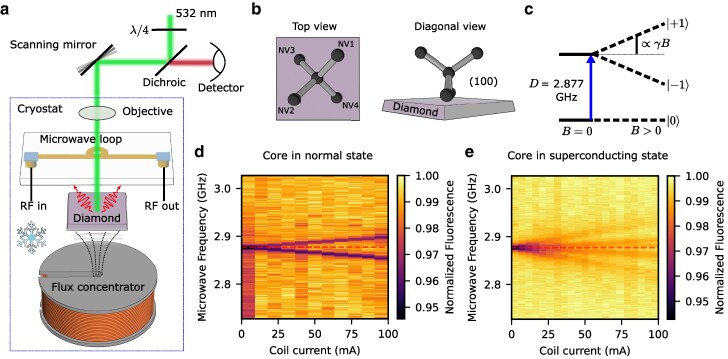
Magnetometry with NV centers. a) A schematic of the measurement setup and configuration. b) The bond orientations of the lattice of a (100) cut diamond. c) The projection of a magnetic field on the NV axis will split the two spin states with $m_{s}=\pm 1$, while their relative frequency with respect to the $m_{s}=0$ state can be read out as an ODMR signal. d, e) Such ODMR spectra plotted as a function of coil current for a core in normal state (d) and in superconducting state (e), where a dashed line represents the center frequency determined by the zero-field splitting *D* at 2.877 GHz.

Here, *D* represents the zero-field splitting, originating from the dipolar interaction between the two unpaired electrons, which is 2.877 GHz in the low-temperature, zero-phonon limit (33). The term *γ* is the electron gyromagnetic ratio of ∼28 GHz/T, describing the Zeeman interaction between the electron spin and an external magnetic field *B*. The Zeeman interaction lifts the degeneracy between the |−1⟩ and |+1⟩ states (Fig. 3c), with the resulting energy splitting providing a direct measure of the magnetic field component along the NV axis (34). See also Supplementary Section S2 for more information about the spin state’s splitting with magnetic field. In our experiment, a diamond sample (ThorLabs DNVB-1, 300 ppb NV concentration, size (*l* × *w* × *h*): 3 mm × 3 mm × 0.5 mm) containing an ensemble of NV centers in four crystallographic orientations was used (Fig. 3b). The diamond is cut along the (100) plane, ensuring that an upward-directed magnetic field projects equally onto all four NV axes.

For magnetic field measurements near the coil, we placed the diamond between the flat side of the flux concentrator and a microfabricated microwave stripline antenna (sputtered gold on chromium sticking layer). This transparent loop-on-glass antenna (see Fig. S5) allows simultaneous excitation of the NV centers with 532 nm laser light (turned to circularly polarized by *λ*/4 plate to excite all populations equally) and collection of their fluorescence through a microscope objective (10×, 0.25 NA) immersed in liquid helium in a cryostat (see Fig. 3a for the setup schematic). Because we can control the cryostat temperature, it is possible to operate in a regime where the niobium core remains in its normal state while the niobium–titanium coil wiring is already superconducting—owing to their differing critical temperatures.

While tuning the coil current up to 100 mA, we recorded ODMR spectra near the surface of the microwave loop at 0.5 mm height from the flux concentrator core. The resulting heatmap of these series of ODMR spectra shows a linear Zeeman shift of 0.013 ± 0.001 mT/mA (Fig. 3d). This value agrees well with finite-element simulations of the core in its normal state (∼0.011 mT/mA). For the normal state measurement, given the large size of the outer coil, the field is homogeneous in the sampling area, which is determined by the microscope objective’s point spread function, offering an in-plane resolution of ∼10 μm. To improve depth discrimination and suppress inhomogeneous ODMR broadening at high fields, a 75 μm pinhole was inserted into the detection path—a standard confocal microscopy practice (see Fig. S7). We estimate the out-of-plane resolution to be in the order of ∼50 μm (see Supplementary Section S2).

Upon lowering the temperature below the critical temperature of niobium, the behavior of the magnetic field changes (Fig. 3e). The ODMR spectra reveal unequal splittings for the four NV orientations, indicating significant field curvature even at this distance above the slit, a result from the strong confinement of the magnetic field to the narrow slit region (see Fig. S8b). The measured Zeeman splitting for the superconducting core, as determined from the NV orientation that most aligns with the field, is 0.043 ± 0.005 mT/mA—about 3.3 ± 0.4 times higher than in the normal state. This enhancement slightly exceeds simulation predictions, likely due to the NV centers residing within a range of heights lower than the nominal 0.5 mm plane.

**Fig. 4.**
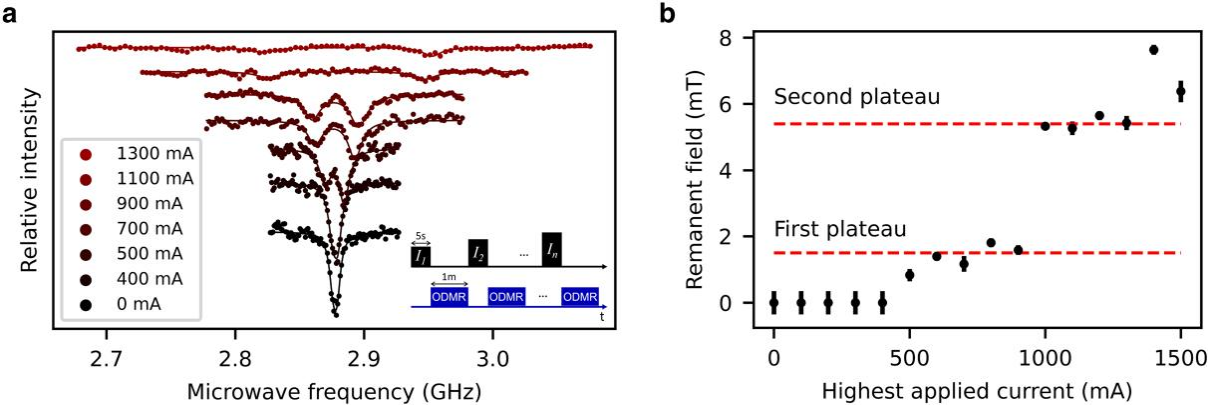
Remanent fields in the flux concentrator after high-current operation. a) Six ODMR spectra taken after applying the labeled amount of current for 5 s (see also the inset measurement scheme). b) The single spectra (including the subset in (a)), have been fitted to two Lorentzian curves, resulting in an estimate for the remanent magnetic field magnitude when solved for Eq. 1. The remanent field has been plotted as a function of the highest applied current to the flux concentrator.

To investigate flux trapping, we applied incrementally increasing coil currents, holding each value for 5 s, then reducing the current to zero and finally recording an ODMR spectrum. This process was repeated in 100 mA steps up to 1.5 A. As shown in Fig. 4a, the ODMR spectrum does not measure a significant field up to 400 mA. However, above this current, a small remanent field has developed, which remained stable until ∼900 mA, beyond which it increased sharply again. At 1 A, a remanent field of ∼5.5 mT persisted at 0.5 mm above the core—comparable to the field generated during continuous operation at 130 mA. This remanent field likely results from flux trapping in the niobium core when the local magnetic field exceeds the lower critical field ($B_{c,1}$). Furthermore, the equivalent projection of the remanent field onto all NV axes reveals that there is little to no curvature in the field as opposed to the current-induced field in the flux concentrator. This could indicate that the remanent field is created by flux penetration and trapping deeper into the core. About a millimeter further outwards of the flux concentrator’s center, we measure a significantly weaker remanent field, indicating the remanent field mostly concentrates in the center of the loop, as expected from the localized breaching of the lower critical field.

## Discussion

The flux trapping effect offers a plausible explanation for several phenomena observed in the levitation experiments. Notably, the levitating particle’s resonance frequency varied by up to 10% between measurements performed with identical current parameters but following different histories of applied current. This is consistent with the percentage of remanent field from prior high-current operation. The presence of a remanent field could also explain why the observed mode frequencies in Fig. 2a are higher than expected from the simulations. Moreover, trapped flux is a known source of damping for levitating magnets or superconducting particles (31, 35, 36) and likely accounts for the sharp drop in quality factor succeeding high-current application. Strong remanent fields and the close proximity of the superconducting core to the levitating particle likely make flux trapping a prominent damping mechanism in this trap.

While simulations predicted that local fields should reach 350 mT at 1.7 A—with the lower critical field ($B_{c,1}=173.5$ mT (30)) being breached around 0.85 A (coinciding with the value of current in Fig. 2b at which quality factors start decreasing)—the first experimental signs of flux trapping appeared between 0.4 and 0.5 A (first plateau in Fig. 4b). Although the calculated field at 0.5 A was ∼100 mT (assuming ideal smooth edges), real-world machining imperfections in the brittle niobium likely enhanced local field concentrations at rough edges, triggering earlier flux penetration. Moreover, taking into account the temperature of 1.4 K (versus 35 mK for the levitation experiments), the temperature-corrected critical field of niobium is lowered to 130 mT and accounts for the earlier onset of flux penetration in the NV magnetometry measurements. The pronounced increase in trapped flux observed between 0.9 and 1 A may reflect a more widespread critical field breach around the entire loop, capacitated by its sharp edges (second plateau in Fig. 4b).

To prevent premature flux trapping, future designs might employ a flux concentrator made from a more malleable material, later polished mechanically or chemically and coated with a high-purity superconducting layer. Since no remanent field was detected up to 400 mA, this may define a safe operating range. Reducing the vertical separation between the coils could further increase the field gradient without elevating the local field at the sensitive core edges. For example, decreasing the separation from 1.2 mm to 200 μm for the same 50 μm particle would raise the vertical field gradient from 56 to ∼175 T/m per 1 A of current, allowing for 60 Hz per 1 A for the x-mode frequencies and >150 Hz for the z-mode frequencies (see Fig. S10). We note that, although no remanent magnetic field is detected up to 400 mA, some magnetic flux may still be trapped in both the flux concentrators and the superconducting particle. This flux trapping can occur during cooldown in presence of background magnetic fields and may limit the observed quality factor at low coil currents. Furthermore, flux freezing in the superconducting particle was clearly observed when the particle temporarily lost superconductivity due to optical heating, fell, and subsequently rethermalized while the coils remained energized. In these cases, the particle no longer levitated and instead behaved as a weak magnet. Heating the particle with a strong laser intensity, while simultaneously switching off the coils successfully removed the trapped flux. In future experiments, flux freezing by background magnetic fields could be mitigated by surrounding the setup with magnetic shielding.

Another alternative is the use of a type I superconductor to avoid flux trapping, but the critical field that can be achieved is significantly lower at 80.3 mT for lead (37). Finally, we emphasize that on-chip flux concentrators could be an appealing alternative to planar on-chip coils, potentially with flip-chip designs (38). Such a design would also allow significant downscaling of the flux concentration region, resulting in increased magnetic field gradients and thus stiffer trapping potentials with vibrational eigenfrequencies into the kilohertz regime. Moreover, the ability of depositing high-purity thin films of superconductors could reduce available defect sites for flux trapping.

## Conclusion

We have demonstrated the design and operation of a superconducting flux concentrator trap capable of levitating superconducting microparticles at millikelvin temperatures. However, during levitation experiments, we observed shifts in the particle’s vibrational resonance frequencies and a marked reduction in quality factors following the application of higher coil currents. These effects suggested the presence of persistent, remanent magnetic fields within the trap, likely caused by flux trapping in the superconducting core once local fields exceeded the lower critical field threshold.

With NV center magnetometry, we confirmed the onset of flux trapping above 500 mA of applied current, with remanent fields persisting after current removal and increasing sharply around 0.9–1.0 A. These observations correlated with both the shifts in levitating particle dynamics and the significant drops in quality factors.

Based on these results, we propose that future flux concentrator designs should consider alternative materials, improved surface treatments or on-chip designs to mitigate premature flux trapping. This work highlights both the capabilities and limitations of superconducting flux concentrator traps and illustrates the power of NV-based magnetometry for diagnosing and optimizing complex cryogenic magnetic systems.

## Supplementary Material

pgag072_Supplementary_Data

## Data Availability

The data that support the findings of this study are available from the corresponding author upon reasonable request, but will not be made available in a persistent repository due to the size of the dataset, which includes high framerate video material.

## Preprints

A preprint of this article is published at 10.48550/arXiv.2507.20795.
