# Supplementary material for: Superconducting flux concentrator coils for levitation of particles in the Meissner state
Source: PNAS Nexus. 2026 Mar 16;5(3):pgag072. doi: 10.1093/pnasnexus/pgag072 (PMC13017030; doi:10.1093/pnasnexus/pgag072)
Supplement: pgag072_Supplementary_Data [file pgag072_supplementary_data.pdf]

Superconducting flux concentrator coils for levitation of particles in the Meissner state: supplementary material

Robert Smit,[1] Martijn Janse,[1] Eli van der Bent,[1] Thijmen de Jong,[1]
Kier Heeck,[1] Jaimy Plugge,[1] Tjerk Oosterkamp,[1] and Bas Hensen[1,*]

[1] *Leiden Institute of Physics, Leiden University, P.O. Box 9504, 2300 RA Leiden, The Netherlands*

(Dated: June 2025)

I. MAGNETIC LEVITATION

The levitation experiments were carried out in a dilution refrigerator from Leiden Cryogenics (model LC CF-CS110), which achieves a standard base temperature of approximately 10 mK. A schematic of the experimental setup within the cryostat is shown in Figure S1. When the maximum illumination intensity from a red LED is coupled into the optical fiber (around 35 μW), the temperature rises modestly and stabilizes between 30 and 35 mK.

The currents for the levitation coils are supplied by two programmable Tenma power supplies. No additional filtering or current stabilization was implemented in our setup, so the motional resonance frequencies are likely broadened by spectral diffusion caused by current noise. To mitigate such fluctuations and achieve higher quality factors, Hofer *et al.* employed a low-pass filter with a sub-Hz cutoff frequency alongside feedback control on the current source output[1]. In our case, for the lower quality factors measured, current noise is likely not the primary limiting factor, as higher Q values have been observed in standard superconducting coil levitation experiments that

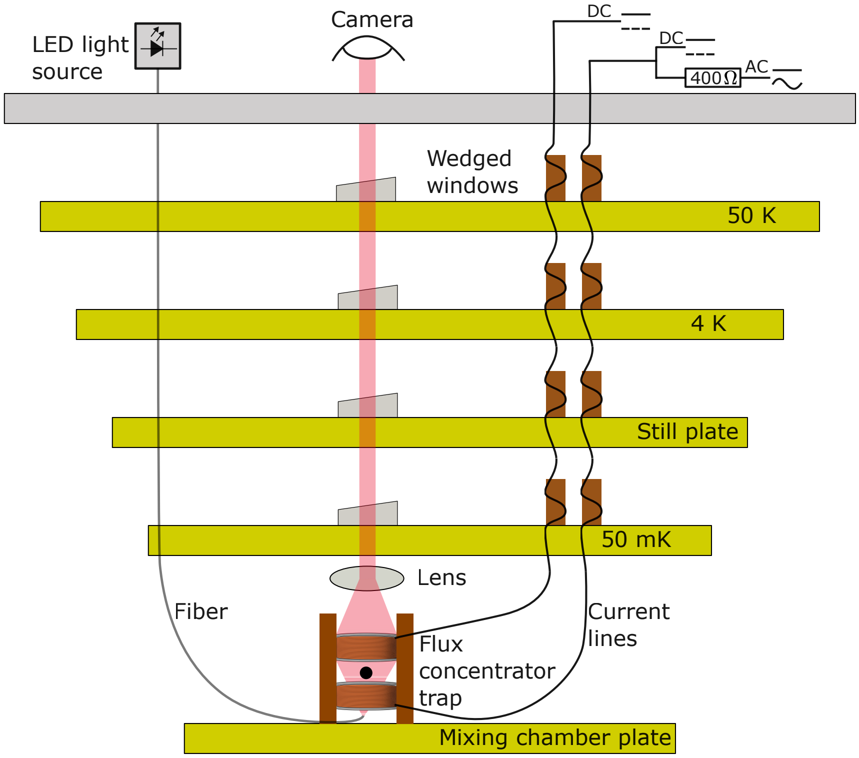

FIG. S1. Schematic of the dilution refrigerator setup with an optical viewport used for the levitation experiments.

* hensen@physics.leidenuniv.nl

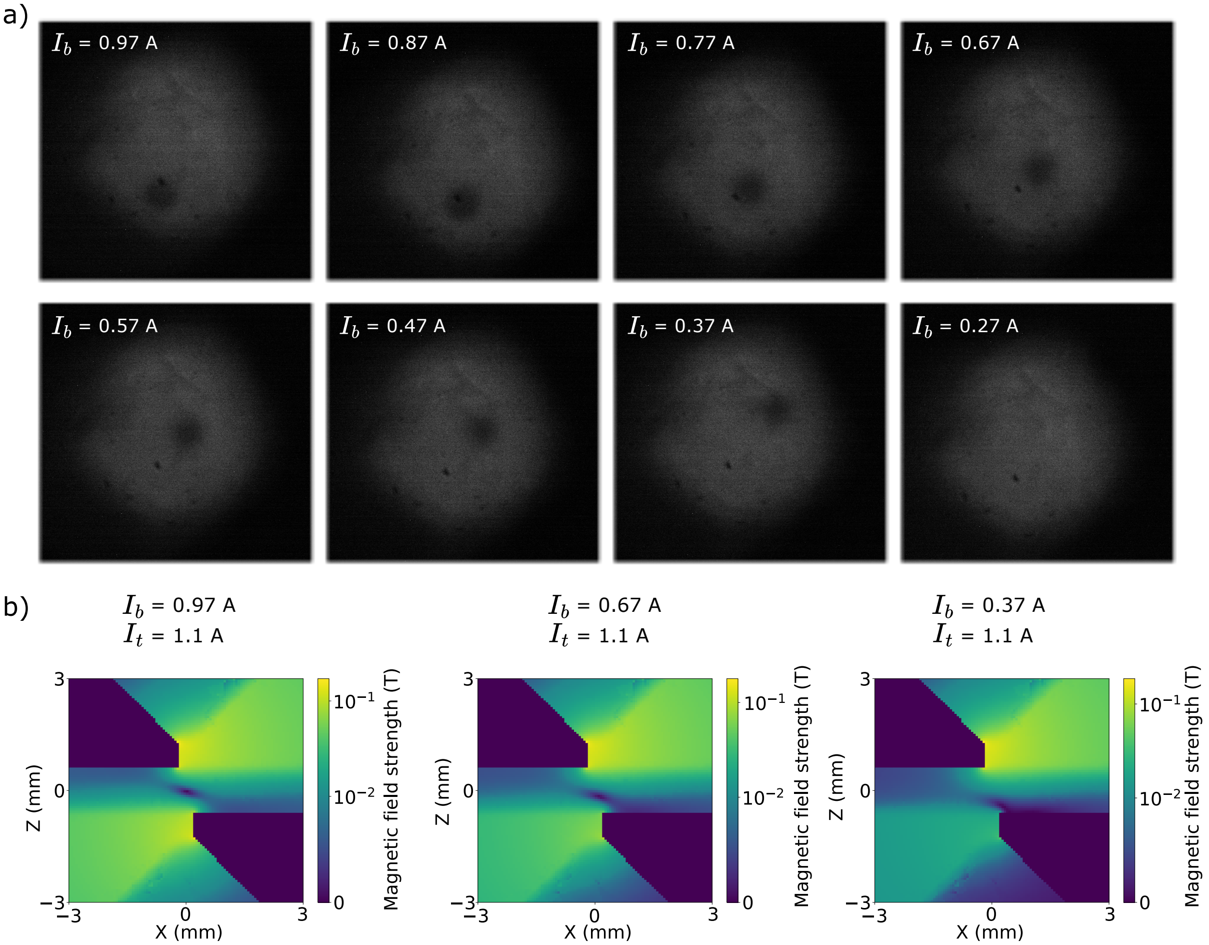

FIG. S2. The top part shows eight captures of the trap center while a fixed top coil current of 1.1 A and a variable bottom coil current, shown as I_b, are applied. The particle's position clearly shifts along the slit direction due to the asymmetry between the two coil currents. At the lowest bottom coil current of 0.27 A the particle is not trapped anymore. Corresponding simulations of the shifting potential minimum for three of those bottom coil currents are shown at the bottom.

we performed under similar conditions.

Before cooling down the cryostat, we position a lens above the flux concentrator trap to ensure proper focus. Light is collected through a series of fused-silica windows mounted on each temperature stage of the cryostat. These windows block thermal radiation while transmitting visible light and are wedged to prevent interference effects such as etaloning. The collected light is then focused onto a CS505MU camera, with image acquisition performed using ThorCam software or through a python script. By optimizing the acquisition parameters, frame rates of up to 300 fps are achieved — more than sufficient for resolving the slit mode.

Examples of recorded trap images are presented in Figure S2. In these image sequences, the current in the top coil is held constant while the current in the bottom coil is varied to increase the asymmetry between the two. As shown by the simulations in the lower part of Figure S2, due to the shape of the trapping potential, the particle's motion involves both in-plane displacement (well resolved in the top-view imaging) and out-of-plane movement (which is less discernible due to limited depth resolution).

For dynamic measurements such as ringdowns, we optimize video acquisition by first driving the particle at its resonance frequency for a set period, then ceasing the drive while continuing to record the motion during the ring-down phase. Each video frame is analyzed by fitting the particle's center position, providing a time series of its motion. This data reveals clear oscillatory behavior and a subsequent decay in amplitude. We fit these time series to an exponentially decaying sinusoid, characterized by a resonance frequency f_0 and a decay time constant τ. The

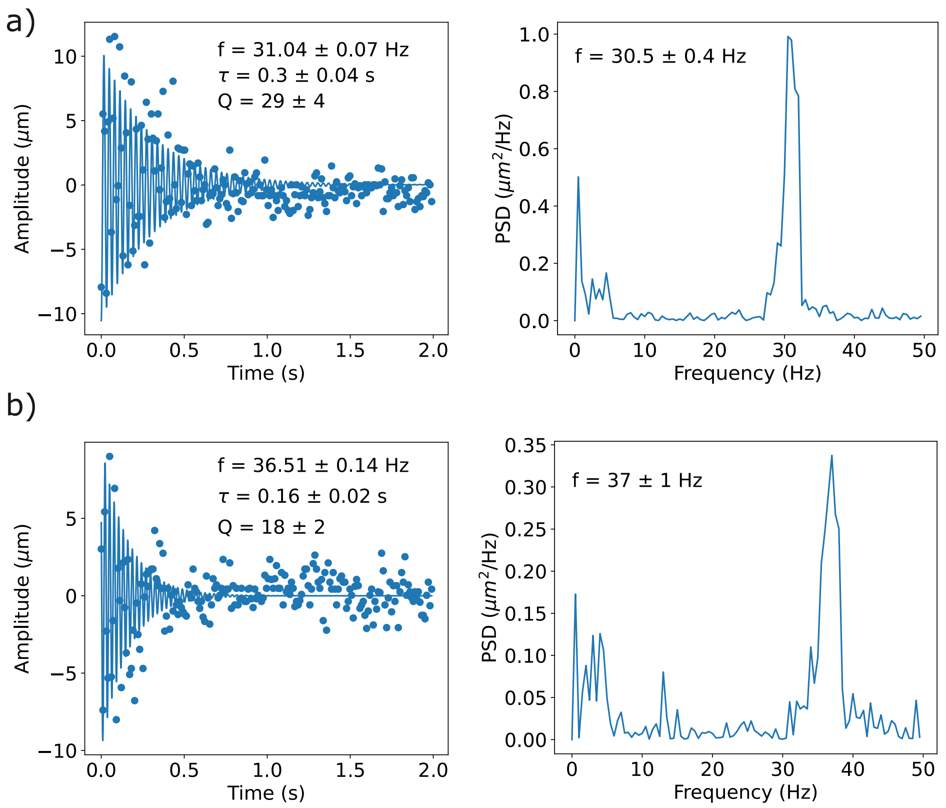

FIG. S3. Ringdown measurements and power spectral density curves obtained with video analysis for different currents. The currents for the bottom and top coil were respectively 1.2 A and 1.6 A in (a) and 1.4 A and 1.7 A in (b).

mechanical quality factor is then calculated using $Q = \pi f_0 \tau$. Two representative examples of these measurements are shown in Figure S3.

II. NV CENTER MAGNETOMETRY

A. Theory

The nitrogen-vacancy (NV) center spin in diamond is an effective probe for detecting magnetic fields, owing to its efficient optical readout via fluorescence. Transitions between the electron spin states $|0\rangle$ and $|\pm 1\rangle$ can be observed through optically detected magnetic resonance (ODMR), appearing as dips in the fluorescence signal with a contrast of up to 30%. In the absence of a magnetic field, both electron spin resonances occur at 2.877 GHz (at 0 K) and are degenerate. When a magnetic field is applied along the NV center axis, the $|-1\rangle$ state shifts to lower frequencies, while the $|+1\rangle$ state shifts to higher frequencies. The resulting splitting between these levels serves as a direct measure of the local magnetic field.

Magnetic field sensing can be performed using a single NV center or an ensemble within a diamond crystal. In a single-crystal diamond, NV centers naturally occur along four crystallographic orientations, determined by the diamond lattice structure. By analyzing the magnetic response of the NV centers along each of these four orientations, it is possible to reconstruct the full magnetic field vector. If the NV axis is taken as the principal z for each orientation, the Hamiltonian describing the system is given by:

$$H/\hbar = D S_z^2 + \gamma \mathbf{B} \cdot \mathbf{S}. \quad (1)$$

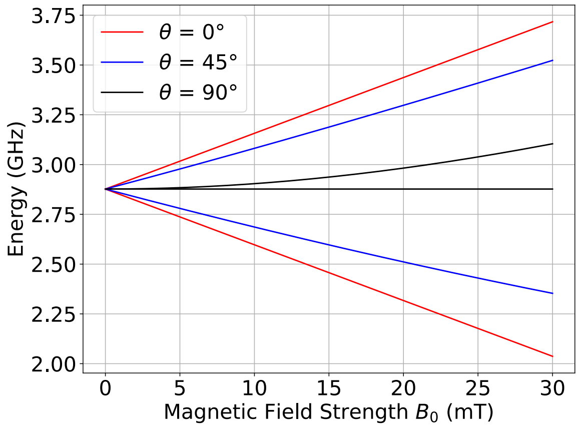

FIG. S4. Energy eigenvalues for the $|-1\rangle$ and $|+1\rangle$ states as a function of magnetic field strength, calculated by solving equation 1 with angular dependence of the field given by equation 3. The three colors represent values for which the magnetic field is along the NV axis (red line), orthogonal to the NV axis (black line) and at an in-between angle (blue line).

Here, D is the zero-field splitting of 2.877 GHz, γ the gyromagnetic ratio and S_z and $\mathbf{S} = (S_x, S_y, S_z)$ are Pauli matrices:

$$S_x = \frac{1}{\sqrt{2}} \begin{pmatrix} 0 & 1 & 0 \\ 1 & 0 & 1 \\ 0 & 1 & 0 \end{pmatrix}, S_y = \frac{1}{\sqrt{2}} \begin{pmatrix} 0 & -i & 0 \\ i & 0 & -i \\ 0 & i & 0 \end{pmatrix}, S_z = \begin{pmatrix} 1 & 0 & 0 \\ 0 & 0 & 0 \\ 0 & 0 & -1 \end{pmatrix}. \tag{2}$$

To determine the local magnetic field, this Hamiltonian must first be diagonalized to obtain the energy eigenvalues of the three spin states. Since the magnetic field can be characterized by an angle θ relative to the NV axis, all combinations of field components in the x and y directions that produce the same angle form a degenerate cone in space. For simplicity, the magnetic field vector can be expressed as:

$$\mathbf{B} = B_0(sin\theta\hat{x} + cos\theta\hat{z}). \tag{3}$$

When the magnetic field is aligned with the NV axis ($\theta = 0$), the splitting of the spin levels is linear, as illustrated by the red line in Figure S4. As the angle increases, the splitting becomes nonlinear, and asymmetries relative to the zero-field splitting become more pronounced at higher magnetic fields (as shown in Figure S7). Furthermore, due to spin state mixing at elevated fields, a measurable splitting persists even when the field is perpendicular to the NV axis.

In measurements involving the flux concentrator's magnetic field, contributions from all four NV orientations must be considered, each with its distinct resonance response as shown in Figure S4. The diamond used in this experiment is cut along the (100) crystallographic plane, making the in-plane and out-of-plane directions symmetry axes. In this configuration, the out-of-plane direction forms an angle of approximately 54.7° with each NV axis, while the in-plane direction is at an angle of 35.3°. This geometry ensures that for out-of-plane magnetic fields — which is typically the case in our setup — the field components project equally onto all four NV orientations.

B. Experiments

A schematic of the NV magnetometry setup is shown in Figure 3 of the main text. The system is built around a confocal fluorescence microscope integrated with a liquid-helium cryostat (Janis SVT), capable of reaching base temperatures of approximately 1.4 K. To drive spin transitions, we fabricated a microwave coplanar waveguide featuring a 400 μm diameter loop on single-side ITO-coated glass (see Figure S5), patterned using electron-beam lithography. To prevent charging during the patterning, a 5 nm chromium layer was deposited on the non-conductive side of the glass, which was later removed via a chromium etch. The microwave structures, consisting of 5 nm chromium / 100 nm gold, remained intact after etching.

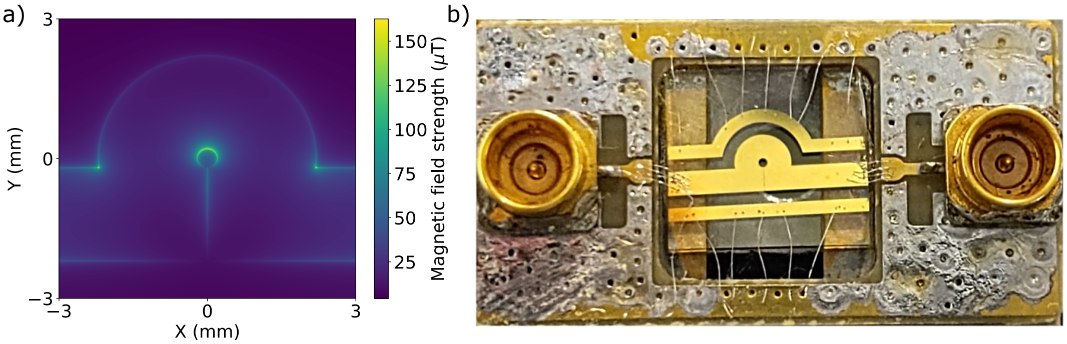

FIG. S5. Panel (a) shows a simulation of the RF magnetic field B_1 in the plane of the microwave loop when the maximum expected current at 25 dB of RF power runs through the loop. The field in the center corresponds to about 50 μT and would give rise to at most 1.4 MHz Rabi flopping rate $\Omega = \gamma B_1$. The corresponding microwave loop used in the experiments is shown in panel (b).

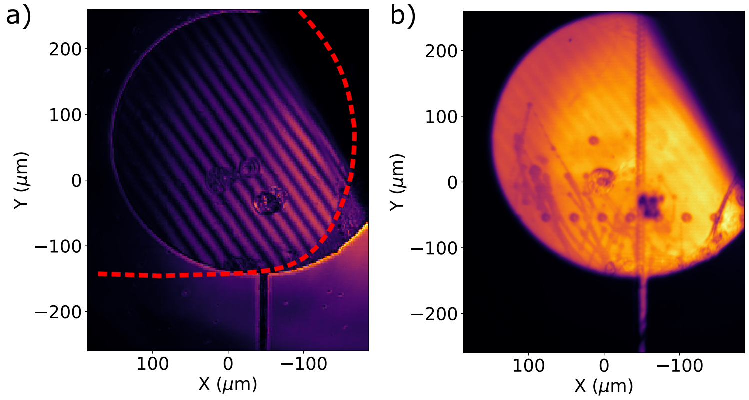

FIG. S6. Confocal scans of reflected light in (a) and fluorescence in (b). The orientation of the inner loop of the flux concentrator is outlined with the dashed line. The slit of the flux concentrator is oriented to the left side of the image. The darker region in the upper right corner is due to a break in the glass of the microwave loop during cooldown.

The processed glass was mounted on a sample holder and connected to a PCB using wire bonds. This was further interfaced with an RF source (R&S SGS100a) capable of delivering up to 25 dB of RF power. The microwave loop was designed for a characteristic impedance of 50 Ω, with measured transmission losses (S_{21}) of only 2–4 dB across a 2–4 GHz frequency range. To achieve optimal ODMR contrast, which reached a maximum of 10%, the microwave signal was pulsed for a few microseconds with a duty cycle of 10%. This approach reduced heat dissipation in the microwave loop, preventing helium boiling and minimizing fluorescence signal fluctuations.

The diamond was imaged through the microwave loop, so fluorescence was collected only from the region within the loop (see Figure S6b). An objective lens (10x, 0.25 NA from Melles Griot) immersed in liquid helium collected the emitted fluorescence, which was then directed through a dichroic mirror and additional optical filters before detection by a single-photon counting module (SPCM-AQRH-14). The detected signal was subsequently processed by an Adwin Gold control system.

The spatial resolution of the magnetometry measurements was primarily limited by the optical resolution of the microscope, which depends on both the numerical aperture (NA) of the objective and the system's depth of field. To improve the depth of field and mitigate spectral broadening caused by magnetic field gradients, a pinhole (75 μm) was inserted into the detection path. The resulting improvement is clearly illustrated in Figure S7, which compares data acquired with and without the pinhole.

Although the exact lateral resolution of the laser spot wasn't directly measured, an upper estimate can be inferred from Figure S6b. In this image, a vertical line and a series of spots along a horizontal line indicate positions where prolonged measurement led to photobleaching. By examining one of these isolated bleached spots, a fluores-

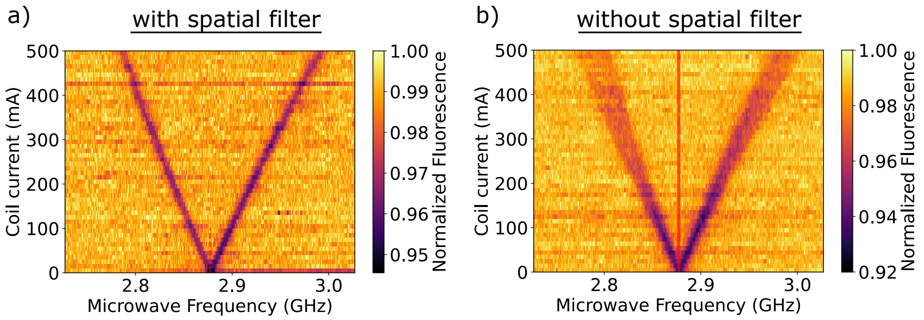

FIG. S7. Series of ODMR spectra for a range of currents through the coil. The spectrum in (a) was taken with a pinhole in the detection path, while the spectrum in (b) was taken without.

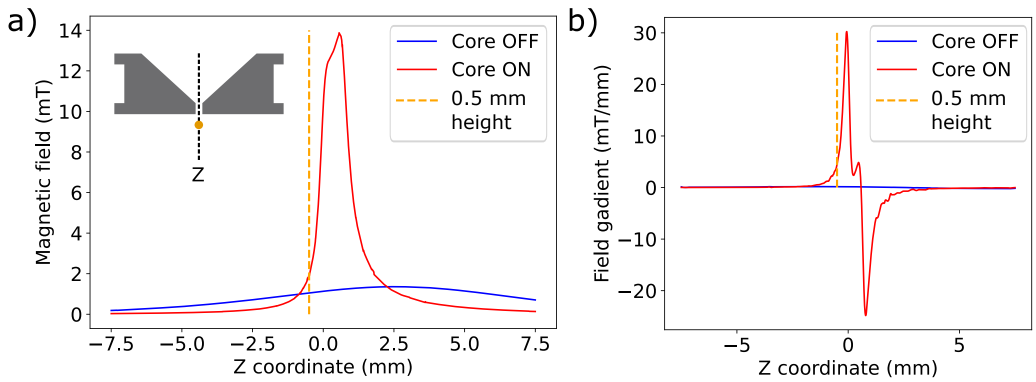

FIG. S8. Panel (a) shows the magnetic field strength in the z direction for a superconducting and non-superconducting core. The inset illustrates how the z coordinate is defined. The ratio of the two maxima is close to 10 - the amplification factor. The position of the maximum in z is different. Panel (b) shows the magnetic field gradient as a function of the z coordinate. The orange dashed line indicates the height at which the magnetic field measurements were taken.

cence bleaching profile with a diameter of approximately 10 μm was determined — about four times larger than the diffraction limit for a 0.25 NA objective at 532 nm, which corresponds to a 2.6 μm beam waist.

Estimating the axial resolution is more challenging, but theoretically it can be expressed as: $\Delta z = 2n\lambda/NA^2$. Given the high refractive index of diamond (n = 2.4), the axial resolution for the 0.25 NA objective is at least 41 μm. This limited axial resolution makes measurements susceptible to magnetic field gradients, which were calculated for the flux concentrator in Figure S8, both with and without a superconducting core, at a driving current of 100 mA. At the measurement height of 0.5 mm above the flux concentrator loop (corresponding to z = -0.5 mm in Figure S8), the magnetic field gradient was approximately 0.166 mT/mm with the core in the normal state, increasing to 4.45 mT/mm in the superconducting state.

The corresponding ODMR spectra reflected these gradients: with a normal-state core (weak gradient), the observed linewidth was about 8 $\pm$ 2 MHz, while with a superconducting core at 100 mA, the linewidth broadened to 28 $\pm$ 4 MHz. The measured field strength of 3 $\pm$ 0.3 mT, combined with the observed linewidth increase, indicated a local field gradient of approximately 0.22 $\pm$ 0.02 mT/mm. From this, the effective axial resolution was estimated to be around 50 μm — slightly larger than the theoretical limit but consistent with the gradient-induced broadening effects. Future optimizations of the axial resolution can include the use of thinner diamonds, together with an integrated microwave antenna. This would allow for the use of higher NA optics with shorter working distances, placed in the cryogenic environment. With an improved optical resolution, the specific locations where flux is trapped can be studied with higher spatial certainty.

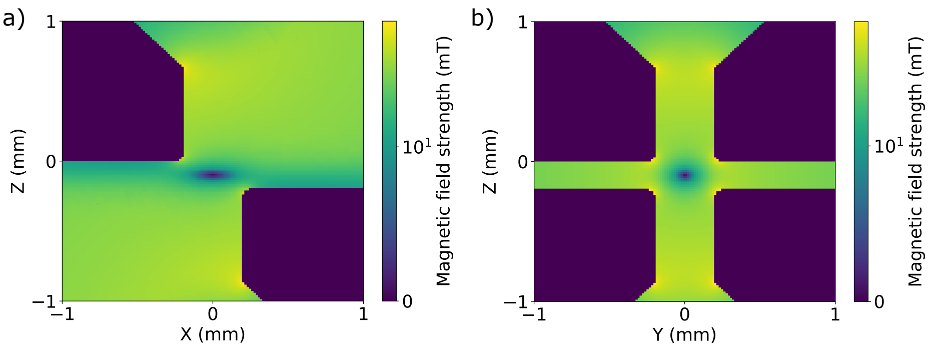

FIG. S9. Simulations of the XZ plane (a) and YZ plane (b) of the anti-Helmholtz field for a configuration in which the radius of the flux concentrator loop matches the vertical separation between the coils.

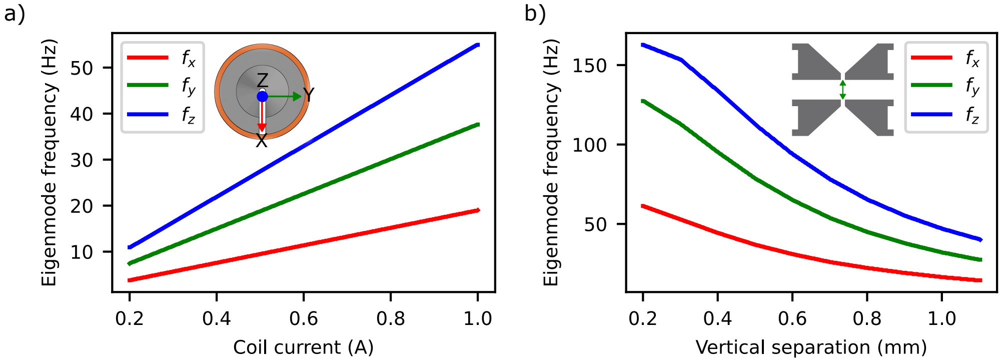

FIG. S10. Simulated eigenfrequencies for the three eigenmodes as a function of coil current in (a) and as a function of vertical separation between the coils in (b). The values in (b) correspond to a simulated current of 1 A through both coils.

III. OPTIMIZATION OF THE FLUX CONCENTRATOR TRAP

With simulations of the flux concentrator trap, we find that the optimal symmetry of the potential minimum is reached when the radius of the inner loop of the flux concentrator matches the spacing between the individual flux concentrators (Figure S9). The further decrease of the vertical separation between the flux concentrators, while keeping currents fixed, increases the eigenmode frequencies, as shown in Figure S10b. The increase of the eigenmode frequencies scales non-linearly with vertical separation and indicates that also the geometric factors of the trap are increasing and thus become more close to those found for an ideal anti-Helmholtz configuration[2].

REFERENCES

[1] J. Hofer, R. Gross, G. Higgins, H. Huebl, O. Kieler, R. Kleiner, D. Koelle, P. Schmidt, J. Slater, M. Trupke, K. Uhl, T. Weimann, W. Wieczorek, and M. Aspelmeyer, High-Q Magnetic Levitation and Control of Superconducting Microspheres at Millikelvin Temperatures, Physical Review Letters **131**, 043603 (2023).

[2] J. Hofer and M. Aspelmeyer, Analytic solutions to the Maxwell–London equations and levitation force for a superconducting sphere in a quadrupole field, Physica Scripta **94**, 125508 (2019).
